# Explainable-enhanced AI for diagnosing coronary microvascular dysfunction with multimodal imaging

**DOI:** 10.1016/j.isci.2025.114101

**Published:** 2025-11-19

**Authors:** Guodong Wang, Lina Guan, Shiyu Li, Yongde Qin, Yunling Wang, Xiaohong Li, Jie Chen, Yuming Mu

**Affiliations:** 1 Department of Echocardiography, The First Affiliated Hospital of Xinjiang Medical University, Urumqi 830000, China; 2 Xinjiang Key Laboratory of Ultrasound Medicine, Urumqi 830000, China; 3 Department of Nuclear Medicine, The First Affiliated Hospital of Xinjiang Medical University, Urumqi 830000, China; 4 Department of Imaging Center, the First Affiliated Hospital of Xinjiang Medical University, Urumqi 830000, China; 5 The Peng Cheng Laboratory, Shenzhen 518055, China; 6 The School of Electronic and Computer Engineering, Peking University, Shenzhen 518055, China

**Keywords:** cardiovascular medicine, artificial intelligence

## Abstract

This study develops a clinically applicable, explainability-enhanced AI model, CMVD_MDAS, to improve the diagnosis of coronary microvascular Dysfunction (CMVD) by integrating deep learning and multimodal machine learning. The model was trained and internally validated on 592 myocardial segments and externally validated on 352 clinical patient segments. The architecture includes automated myocardial segmentation, convolutional neural network-based feature extraction, and multimodal diagnostic modeling with SHAP-based feature ranking to enhance explainability. The CMVD_MDAS demonstrated excellent internal performance (AUC: 0.999) and robust external performance (AUC: 0.79), surpassing physician assessments and commercial software. This explainability-enhanced AI solution significantly improves the accuracy and efficiency of CMVD assessment, reducing diagnostic time by approximately 90% and offering a potential diagnostic tool for clinical practice.

## Highlights

• CMVD_MDAS integrates multimodal AI for accurate, segment-level diagnosis of CMVD
• Explainable AI is superior to physician and commercial software assessment
• AI model boosts efficiency, cutting diagnostic processing time by 90%

## Graphical abstract

**Figure:**
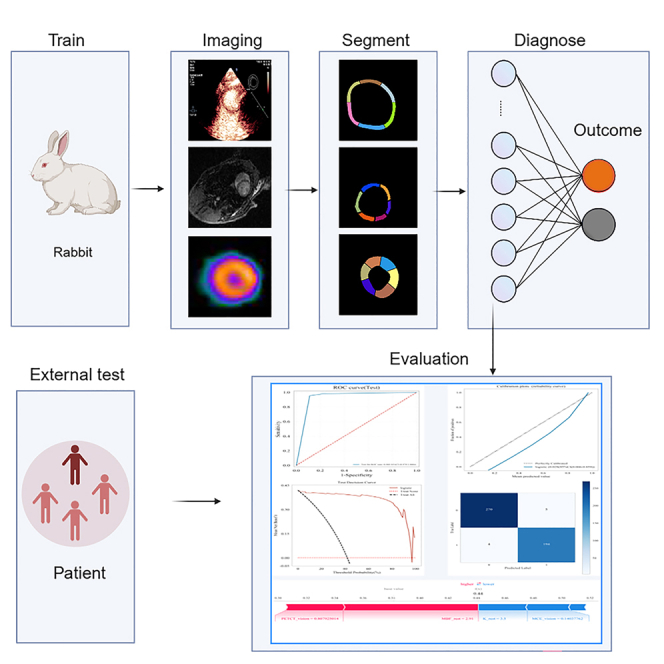

## Introduction

Coronary microvascular dysfunction (CMVD) is characterized by structural and functional impairments of the coronary microvasculature, leading to reduced coronary blood flow (CBF) and ultimately resulting in myocardial ischemia.^1^ Among approximately 112 million patients worldwide experiencing angina,^2^ nearly 50% with angiographically non-obstructive coronary artery disease, as confirmed by coronary angiography, have CMVD.^3^ CMVD is associated with an elevated risk of major adverse cardiovascular events, persistent anginal symptoms, diminished quality of life, and increased healthcare burden.^2^^,^^4^^,^^5^ Given its high prevalence and often protracted asymptomatic phase, early and accurate diagnosis of CMVD is essential to guide appropriate clinical management. However, current assessments are modality-specific and operator-dependent, complicating segment-level diagnosis.

Although invasive coronary Doppler wire measurement of the coronary flow reserve (CFR) remains the reference clinical method for CMVD assessment,^6^ its widespread clinical application is limited by procedural invasiveness, restricted availability of specialized equipment, and technical complexity. Recent advancements in noninvasive imaging modalities—such as echocardiography (Echo),^7^^,^^8^ myocardial metabolism imaging (MMI) using positron emission tomography/computed tomography (PET/CT),^9^ and cardiac magnetic resonance (CMR) imaging^10^^,^^11^—have facilitated both qualitative and quantitative evaluation of CMVD. Most prior CMVD studies interrogate perfusion alone and do not jointly model perfusion, mechanics, and metabolism at the segment level.^12^^,^^13^^,^^14^ Multimodal imaging may provide synergistic insights by integrating real-time myocardial mechanics, perfusion, and metabolic data. However, heterogeneity in imaging formats and dependence on subjective interpretation constrain its routine clinical deployment. Therefore, there is an urgent need for robust data-driven frameworks capable of integrating large-scale multimodal imaging datasets to enable objective, quantitative, and reproducible diagnosis of CMVD.

Deep learning (DL) has achieved state-of-the-art performance in medical imaging tasks, including disease classification, lesion detection, segmentation, and quantification.^15^ DL-based approaches have achieved superior accuracy in myocardial segmentation and lesion classification, conferring benefits for reproducibility and diagnostic objectivity.^16^^,^^17^^,^^18^^,^^19^^,^^20^^,^^21^^,^^22^ Despite these advances, clinical translation is impeded by limited model explainability, as the opaque nature of DL decision-making undermines clinician trust and acceptance.^23^ To bridge this gap, explainability-enhanced AI models now provide predictive power with biologically meaningful and interpretable insights.

This study presents CMVD_MDAS, a multistage explainability-enhanced artificial intelligence system developed for segment-level diagnosis of CMVD based on multimodal imaging. Our contributions are: an explainable multimodal pipeline at AHA 16-segment resolution; a modular design enabling component-wise validation; and translational evaluation from H&E-benchmarked animals to patients. CMVD_MDAS integrates automated myocardial segmentation and multiparametric feature extraction from echocardiography, CMR, and PET/CT, enabling machine-learning-based binary classification to deliver a comprehensive, efficient, and interpretable assessment of coronary microvascular function. By leveraging a rabbit CMVD model benchmarked to histopathology and further externally validated in clinical patient cohorts, CMVD_MDAS aims to demonstrate feasibility, robust diagnostic performance, and clinical applicability, potentially advancing the paradigm of data-driven precision medicine for coronary microvascular diseases.

## Results

### Overview of the study

**Figure 1:**
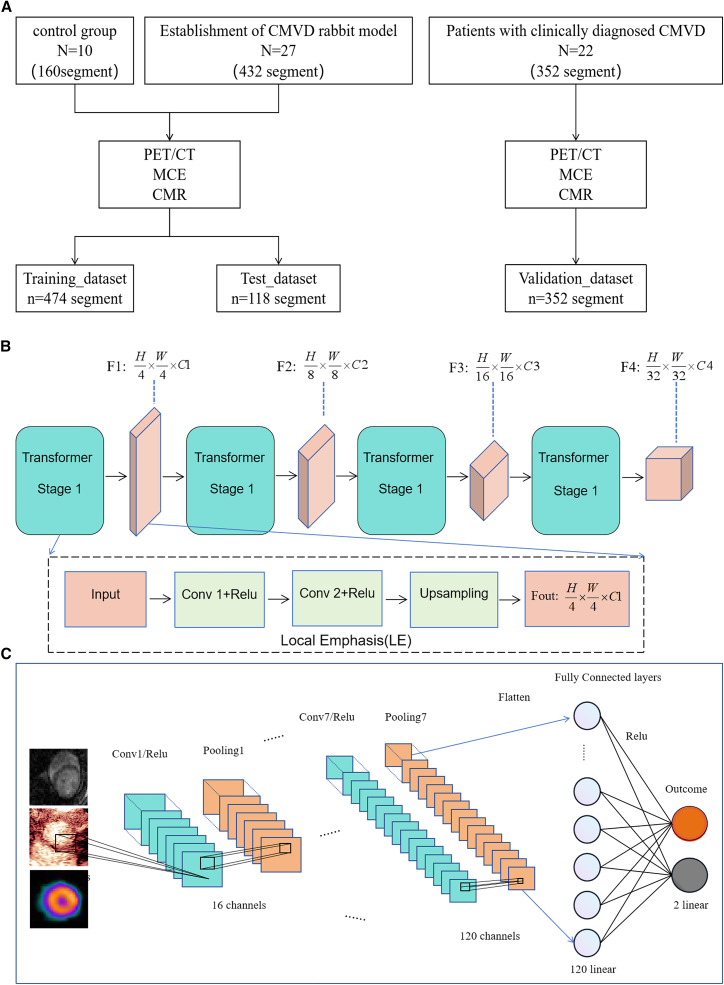
Overview of the CMVD_MDAS data and DL framework (A) Dataset description: CMVD_MDAS was developed using images from a total of 37 New Zealand white rabbit CMVD animal models (592 myocardial segments, 291,884 images), consisting of MCE, CMR, and PET/CT MMI. The generalizability of the model was further evaluated using a clinical dataset of 22 CMVD patients (352 myocardial segments, 20,741 images). (B) CMVD_MDAS segmentation model overview: the segmentation model, based on a transformer framework, outputs myocardial segmentation and segment-level analysis from multimodal cardiac images. (C) CMVD_MDAS classification model: this model provides CMVD diagnosis for both rabbit and human datasets.

In this study, we designed CMVD_MDAS as a multistage and interpretable artificial intelligence diagnostic framework tailored for coronary microvascular disease diagnosis at the myocardial segment level (Figure 1). The model comprises three stages.

#### Segmentation stage

A transformer-based model (SSformer) performs modality-specific myocardial segmentation across echocardiography (MCE), CMR, and PET/CT scans to ensure anatomical accuracy and consistency. This stage enhances explainability by isolating clinically relevant regions for further analysis.

#### Feature extraction and classification stage

Quantitative perfusion parameters (e.g., β from MCE), myocardial strain metrics, and PET-derived metabolic activity are computed and combined with qualitative classification of myocardial perfusion patterns. This fusion allows the model to mimic clinical reasoning by integrating quantitative and visual cues.

#### Diagnostic classification stage

An LGBMClassifier was used for final diagnostic decision. SHapley Additive exPlanations (SHAP) analysis was employed to identify the contribution of each parameter toward model prediction, enabling a transparent interpretation of results. Additionally, heat and attention maps were generated to visualize salient features in the input images.

This staged approach improves modularity, facilitates clinical understanding, and allows separate validation of each functional block, thereby enhancing both diagnostic robustness and explainability.

Based on the 16-segment left ventricular segmentation method proposed by the AHA,^24^ this study included 27 CMVD models and 10 control New Zealand white rabbits, totaling 592 myocardial segments. In total, 291,884 multimodal images were used for model training and internal validation (80%/20% split). For external validation, 352 myocardial segments from 22 patients with CMVD, comprising 20,741 multimodal images, were used. The diagnostic results of CMVD_MDAS were compared with those of expert visual diagnosis and commercial software. A CMVD diagnostic model was developed to address the issues of low diagnostic performance and speed using DL.

### Sample characteristics of study subjects

Study cohorts comprised 944 myocardial segments from animal models and clinical patients. 592 myocardial segments from rabbits, including 344 CMVD-negative and 248 CMVD-positive segments, were used as training and internal validation sets, whereas 352 myocardial segments from patients, including 125 CMVD-negative and 227 CMVD-positive segments, were used as the external validation cohort to assess diagnostic performance and generalizability of CMVD_MDAS. Detailed characteristics of the study participants are provided in Table 1.

**Table 1:** Basic characteristics

	Rabbit (*n* = 37,592 segments)	Patient (*n* = 22,352 segments)
Age	–	49.68 ± 11.38
Sex		
Male	37/37 (100%)	16/22 (73%)
Female	0/37 (0%)	6/22 (27%)
Body weight	3.8 ± 0.13	73.45 ± 12.89
LVEF (%)	73.01 ± 4.80	63.15 ± 4.59
LVFS (%)	39.94 ± 6.49	34.35 ± 3.07
CMVD classes		
Positive segment	248/592 (41.89%)	227/352 (27.5%)
Negative segment	344/592 (58.11%)	125/352 (72.5%)

LVEF, left ventricular ejection fraction; LVFS, left ventricular fractional shortening; CMVD, coronary microvascular disease.

Data are represented as mean ± SEM.

### Evaluation of CMVD_MDAS segmentation model

**Figure 2:**
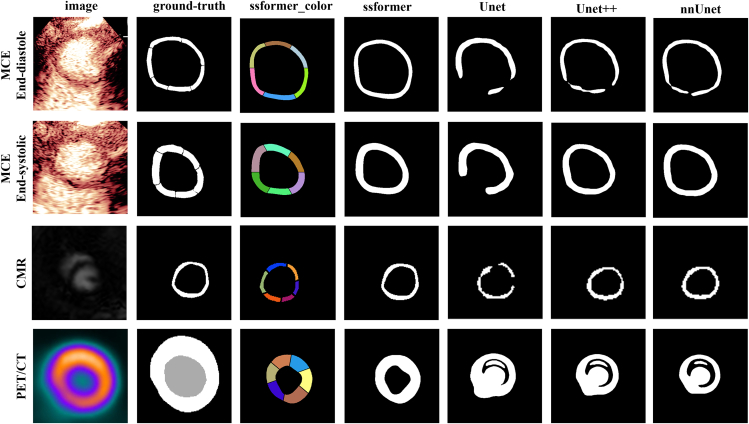
Representative images of CMVD_MDAS segmentation task The first to fourth rows display images of MCE at end diastole, MCE at end systole, cardiac MRI, and ^18^F-FDG PET/CT, respectively. The segmentation results of the SSformer model compared with those of the Unet, Unet++, and nnUnet models are shown through multimodal imaging masks from MCE, CMR, and PET/CT. Myocardial segmentation is shown in the corresponding images.

SSformer achieved superior myocardial segmentation across multimodal images compared to other models. The CMVD_MDAS segmentation model generated segmentation masks for both myocardium and myocardial segments in each image. Because the data analysis of CMVD primarily focuses on end-systole and end-diastole, model segmentation primarily targets these two cardiac phases. In the MCE, CMR, and PET/CT multimodal image datasets, the model segmented both dynamic and static images. Each segment was assigned a distinct color label to improve visualization of myocardial segments. The SSformer segmentation model outperformed Unet, Unet++, and nnUnet models, showing more precise delineation of endocardial and epicardial boundaries and delivering improved boundary accuracy and achieving continuous, complete myocardial segmentation. In contrast, the Unet, Unet++, and nnUnet models exhibited partial discontinuities or drifts in the segmentation masks. Representative images are shown in Figure 2.

**Figure 3:**
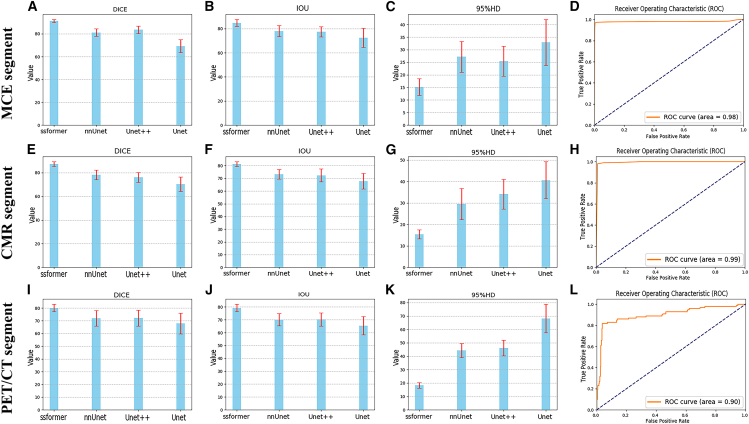
Comparison of segmentation performance among different models This figure compares the segmentation performance of various models (SSformer, Unet, Unet++, and nnUnet) across different imaging modalities, including MCE, CMR, and PET/CT. The results highlight the differences in segmentation accuracy and precision between myocardial and segment boundaries. The short red lines on the bars represent standard deviations. Panels A–D correspond to the MCE modality, E–H to the CMR modality, and I–L to the PET/CT modality, with performance metrics including DICE coefficient, Intersection over Union (IoU), 95% Hausdorff Distance (95% HD), and ROC curve analysis.

In this study, we quantitatively evaluated the performance of four DL models on PET, CMR, and MCE image segmentation tasks. Across multiple evaluation metrics, including precision, recall, the Dice coefficient, F-measure, F-score, intersection over union (IOU), and Hausdorff_95, both Dice and IOU served as key indicators of overlap, whereas Hausdorff_95 assessed the smoothness and accuracy of segmentation boundaries. Through a comprehensive comparative analysis, the SSformer model demonstrated most stable and robust performance, attributable to its pyramid transformer encoder, which enhances generalization capability, along with a progressive local decoder (PLD) that strengthens local feature perception and constrains attention diffusion (Figure 3). In the echocardiographic dataset, SSformer achieved the highest segmentation accuracy, with a Dice coefficient of 0.9153, IOU of 0.8437, and Hausdorff_95 of 22.858, surpassing the results of Unet, Unet++, and nnUnet models, with differences reaching statistical significance. In the CMR dataset, SSformer attained a Dice coefficient of 0.8736, an IOU of 0.8123, and a Hausdorff_95 of 15.42, also surpassing the Unet, Unet++, and nnUnet models with statistically significant differences. For the PET/CT dataset, SSformer achieved a Dice coefficient of 0.8007, an IOU of 0.7923, and a Hausdorff_95 of 18.30, significantly outperforming the other models (see Table S1). This was further corroborated by a quantitative evaluation of attention mechanisms of the model. As described in the main text, the SSformer model, which leverages its advanced transformer architecture coupled with a PLD, precisely allocates computational resources and attention weights, markedly enhancing focus on the target region (myocardium) while effectively suppressing background attention to non-target areas (cardiac chambers). This refined “computational” capability is evident in improved segmentation performance. As shown in attention heatmaps in Figure S4, the model showed high regional specificity and structural recognition accuracy.

### CMVD_MDAS quantitative analysis results

Quantified perfusion and strain parameters showed significant associations with CMVD status. In the rabbit and patient cohorts, 11 and 8 quantitative metrics, respectively, were obtained using commercial software. Specifically, in the rabbit cohort, PET/CT analysis provided the PET_score, SUV_bw_, SUV_heart_liver_, and SUV_heart_lung_; MCE analysis yielded K_rest, A_rest, MBF_rest, MCE_Strain_rest, and TTP_rest; and CMR analysis offered MAX_SIrest and CMR_Strain_rest. Among rabbits, comparisons between positive and negative segments showed no statistically significant differences in SUV_bw_, SUV_heart_lung_, A_rest, and CMR_Strain_rest (*p* > 0.05), whereas PET_score, SUV_heart_liver_, K_rest, MBF_rest, MCE_Strain_rest, TTP_rest, and MAX_SIrest were significantly different (*p* < 0.05).

Using the CMVD_MDAS model, six quantitative parameters, K_rest, A_rest, MBF_rest, MCE_Strain_rest, MAX_SIrest, and CMR_Strain_rest, were automatically obtained. Although A_rest and CMR_Strain_rest were not statistically significant, they remain clinically relevant in the diagnosis of coronary microvascular dysfunction. Therefore, these six parameters were incorporated into the CMVD_MDAS model.

In the patient cohort, comparisons between positive and negative segments revealed no statistically significant differences in K_rest, A_rest, MBF_rest, MCE_Strain_rest, and cine_Strain_rest (*p* > 0.05), whereas MAX_SI showed significant difference, with the negative segment group at 0.878 [0.637, 1.337] and the positive segment group at 0.786 [0.473, 1.155], indicating reduced myocardial perfusion in CMVD-positive segments on CMR. Quantitative analysis using the CMVD_MDAS model demonstrated significant differences in MCE_Strain_rest, MAX_SI, and cine_Strain_rest between positive and negative segments, reflecting reduced perfusion and strain in the positive segments (Table 2).

**Table 2:** Quantitative results of each myocardial segment through software analysis and AI-based analysis

Cohort	Parameter	Software-based quantitative analysis			AI-driven quantitative analysis		
		0 (*N* = 344)	1 (*N* = 248)	*P*	0 (*N* = 344)	1 (*N* = 248)	*P*
Rabbit	PET_score	83.000 [73.000,90.000]	70.000 [53.000,80.000]	<0.001	–	–	–
	SUVbw	2.047 [1.661,2.502]	1.910 [1.571,2.609]	0.153	–	–	–
	SUVheart_liver	1.910 [1.561,2.412]	1.795 [1.423,2.135]	0.001	–	–	–
	SUVheart_lung	3.449 [2.578,4.413]	3.462 [2.670,4.469]	0.655	–	–	–
	K_rest	2.195 [1.730,3.018]	1.930 [1.643,2.467]	<0.001	4.590 [3.530,6.370]	4.230 [3.510,5.170]	<0.001
	A_rest	8.693 [7.435,9.902]	8.693 [7.249,10.644]	0.153	0.680 [0.490,0.860]	0.490 [0.400,0.600]	<0.001
	MBF_rest	18.107 [14.971,24.252]	17.292 [14.383,20.093]	<0.001	2.970 [2.770,3.650]	2.110 [1.980,2.240]	<0.001
	MCE_Strain_rest	24.557 [19.100,29.800]	21.700 [17.800,25.800]	<0.001	0.249 [0.224,0.277]	0.210 [0.158,0.250]	<0.001
	TTPrest	127.000 [115.000,144.000]	133.000 [124.000,150.000]	0.007	–	–	–
	MAX_SIrest	21.617 [16.492,28.215]	25.099 [20.516,29.625]	<0.001	0.718 [0.609,0.850]	0.732 [0.614,0.871]	0.608
	CMR_Strain_rest	12.500 [7.500,19.600]	12.400 [7.600,18.700]	0.639	0.242 [0.214,0.278]	0.120 [0.095,0.150]	<0.001
Patient	K_rest	5.787 [3.505,9.743]	5.196 [2.519,9.106]	0.173	4.810 [3.930,5.490]	4.390 [3.520,5.410]	0.065
	A_rest	9.513 [6.707,13.624]	8.909 [6.208,12.342]	0.251	0.560 [0.410,0.720]	0.550 [0.430,0.700]	0.998
	MBF_rest	52.787 [25.989,112.633]	40.264 [18.034,94.197]	0.121	2.510 [1.970,2.750]	2.580 [2.050,3.290]	0.088
	MCE_Strain_rest	31.493 ± 8.495	30.062 ± 10.840	0.205	0.237 [0.182,0.251]	0.186 [0.176,0.236]	<0.001
	TTP	337.000 [304.000,350.000]	322.000 [300.000,347.000]	0.013	–	–	–
	PET_score	77.000 [69.000,82.000]	65.000 [57.000,70.000]	<0.001	–	–	–
	MAX_SI	0.878 [0.637,1.337]	0.786 [0.473,1.155]	0.011	0.312 [0.252,0.333]	0.249 [0.239,0.306]	<0.001
	cine_Strain_rest	26.400 [19.300,34.400]	27.700 [20.900,34.800]	0.338	0.219 [0.180,0.233]	0.183 [0.174,0.221]	<0.001

Data are presented as mean (95% CI) or median (95% CI). SUV_bw_, standardized uptake value based on body weight; SUV, standard uptake value; MBF, myocardial blood flow; TTP, time to peak; MCE, myocardial contrast echocardiography; PET/CT, positron emission tomography/computed tomography; CMR, cardiac magnetic resonance imaging; CMVD, coronary microvascular dysfunction.

### CMVD_MDAS classification model evaluation and validation

#### Feature selection and importance analysis of influencing factors

**Figure 4:**
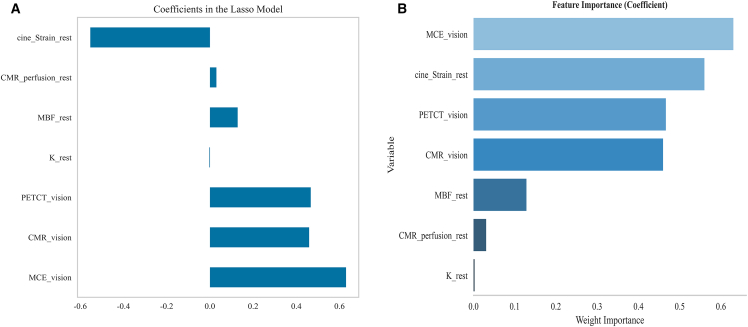
Feature selection results (A) LassoCV method. (B) Variable importance ranking analysis.

Visual assessments and strain parameters were key predictors for CMVD diagnosis. This study selected seven factors for detailed analysis (Figure 4), including three qualitative imaging assessment indicators (“MCE_vision,” “CMR_vision,” and “PETCT_vision”) and four quantitative parameters (“K_rest,” “MBF_rest,” “CMR_perfusion_rest,” and “cine_Strain_rest”). Variable importance was evaluated using the Lasso regression model, which identified “MCE_vision,” “cine_Strain_rest,” and “PETCT_vision” as the top three key predictors contributing most to the diagnosis of coronary microvascular diseases, with importance weights of 0.63, 0.56, and 0.466, respectively.

#### Binary classification multi-model analysis—Optimal model selection

LightGBM demonstrated optimal performance and was selected as the final classifier. This study evaluated the classification performance of several machine learning models, including logistic regression, LGBMClassifier, random forest classifier, and AdaBoost classifier. The ROC curves for each model were obtained using 10-fold cross-validation, and mean AUC values with their standard deviations were calculated.

**Figure 5:**
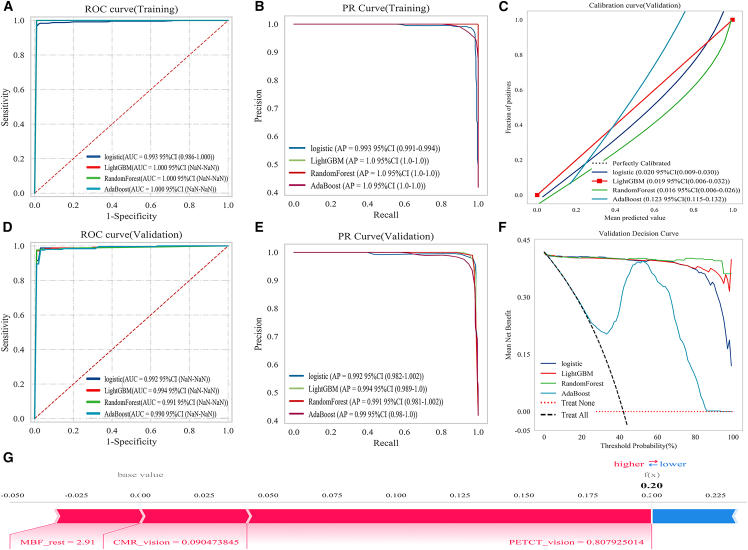
Model performance evaluation and feature contribution analysis (A) ROC curve of the training set, illustrating the discriminative ability of the model. (B) PR curve of the training set, assessing the balance between precision and recall. (C) Calibration curve, evaluating the agreement between predicted and actual outcomes. (D) ROC curve of the internal validation set, demonstrating model performance on unseen data. (E) PR curve of the internal validation set, highlighting predictive performance in imbalanced data scenarios. (F) Decision curve, analyzing the clinical utility of the model. (G) SHAP explanation diagram for a representative rabbit, where the predicted probability of CMVD is 0.20. The features MBF_rest, CMR_vision, and PET/CT_vision contribute positively to the prediction in this sample.

Across models, LightGBM demonstrated the optimum performance, achieving an AUC of 1.000 (95% confidence interval [CI] not available) on the training set and 0.994 (95% CI not available) on the validation set, indicating excellent discriminative ability and model stability. Figures 5A and 5D show the ROC curves, with shaded areas representing variability across folds.

Further evaluation using precision-recall (PR) curves (Figures 5B and 5E), calibration curves, and decision curves (Figures 5C and 5F) confirmed the superior performance and clinical utility of the LGBMClassifier (see Figure S5). All quantitative results were presented with CIs or error bars to reflect the statistical uncertainty and robustness of model predictions.

The SHAP explanation diagram for a representative rabbit shows a predicted CMVD probability of 0.20 (see Figure 5G). In this case, the features MBF_rest, CMR_vision, and PET/CT_vision contributed positively to prediction, indicating greater estimated disease risk. The SHAP values quantify the impact of each feature, with positive values pushing predictions toward CMVD and negative values lowering them. This figure highlights the relative importance of the features and provides decisional interpretability of the model for this sample. The probability of 0.20 indicates a low CMVD risk, consistent with feature contributions. All SHAP values were within physiological or clinical measurement ranges, ensuring meaningful interpretation. This individualized explanation supports segment-level diagnoses and enhances trust in AI systems.

#### External validation of the LightGBM model

**Figure 6:**
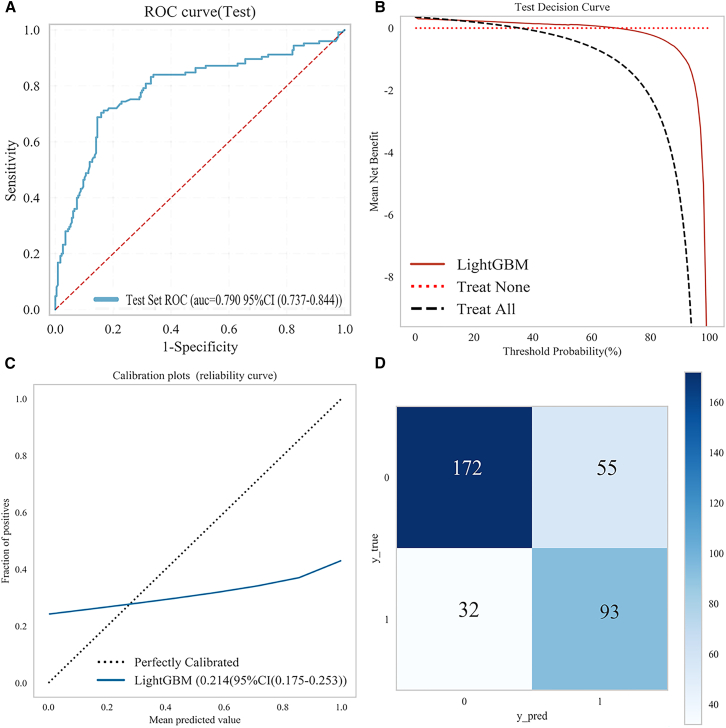
Performance evaluation of the model in the external validation set (A) ROC curve of the external validation set, illustrating the discriminative ability of the model. (B) Decision curve, assessing the clinical utility of the model. (C) Calibration curve, evaluating the agreement between predicted and actual outcomes. (D) A confusion matrix provides a detailed breakdown of the classification performance of the model.

LightGBM maintained good performance in external clinical validation. The LightGBM classification model, initially developed using a rabbit cohort, was externally validated in a clinical patient cohort to evaluate its generalizability and clinical utility. In the external validation set, the model achieved an area under the receiver operating characteristic curve (AUC) of 0.79 with a 95% CI ranging from 0.737 to 0.844, indicating good discriminative ability (Figure 6A). The sensitivity and specificity were 0.704 and 0.841, respectively, indicating a balanced performance in correctly identifying positive and negative cases. Figure 6 shows the ROC curve, with the shaded area representing the variability across folds, emphasizing model stability. Clinical net benefit, reliability, and diagnostic accuracy of the model were assessed using decision curves, calibration curves, and confusion matrix analyses (Figures 6B–6D). These results suggest that the LightGBM model shows robust diagnostic performance when applied to independent clinical data, supporting its potential for clinical translation.

#### Comparison of diagnostic efficiency between CMVD_MDAS, radiologists, and commercial software

**Figure 7:**
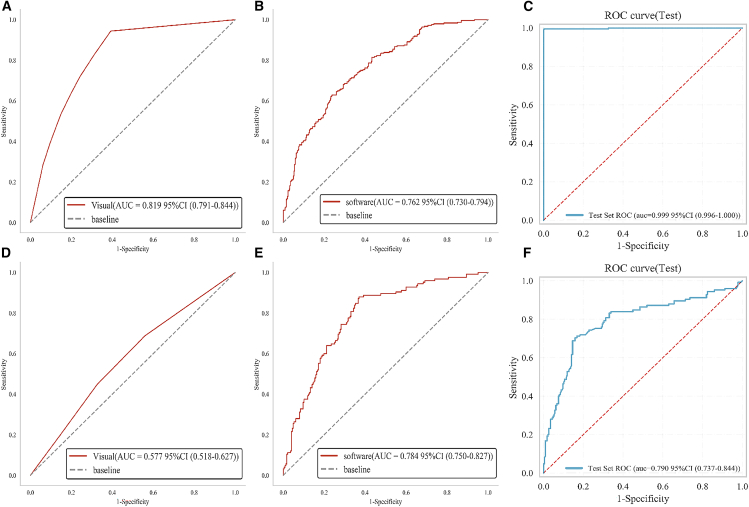
Diagnostic performance of CMVD_MDAS in identifying CMVD abnormal segments in animal and patient cohorts (A–C) Rabbit cohort; (D–F) patient cohort. The ROC curves compare the diagnostic performance of CMVD_MDAS with expert diagnosis and commercial software analysis in CMVD classification.

CMVD_MDAS outperformed radiologists and matched commercial software performance. To evaluate the diagnostic efficiency of CMVD_MDAS compared to that of radiologists and commercial analysis software, performance assessments were conducted on both animal and clinical patient cohorts using multimodal imaging data. In the rabbit cohort (Figures 7A–7C), CMVD_MDAS achieved excellent diagnostic performance, with an area under the ROC curve (AUC) of 0.999 (95% CI: 0.996–1.000), significantly outperforming both expert visual diagnosis and commercial software analysis (*p* < 0.05). The sensitivity and specificity metrics for CMVD_MDAS were also notably higher, underscoring its superior accuracy.

In the clinical patient cohort (Figures 7D–7F), the AUCs of CMVD_MDAS and commercial software were 0.79 (95% CI: 0.737–0.844) and 0.784 (95% CI: 0.750–0.827), respectively, with no statistically significant difference between them (*p* > 0.05). However, both methods significantly outperformed the radiologists’ visual diagnoses, yielding a lower AUC of 0.577 (95% CI: 0.518–0.627) (*p* < 0.05). Moreover, CMVD_MDAS demonstrated higher diagnostic specificity than the commercial software (specificity: 0.84 vs. 0.63), highlighting its greater ability to correctly identify negative cases.

Detailed quantitative metrics, including sensitivity, specificity, accuracy, positive predictive value, negative predictive value, and AUC with 95% CIs for all diagnostic approaches, are summarized in Table 3.

**Table 3:** CMVD_MDAS visual and software performance comparison for rabbit and patient sets

Model	Sensitivity	Specificity	AUC (95% CI)
**Rabbit**			

Visual	0.944	0.608	0.819 (0.791–0.844)
Software	0.629	0.762	0.762 (0.730–0.794)
CMVD_MDAS	0.996	0.997	0.999 (0.996–1.000)

**Patient**			

Visual	0.688	0.441	0.577 (0.518–0.627)
Software	0.880	0.630	0.784 (0.750–0.827)
CMVD_MDAS	0.704	0.841	0.79 (0.737–0.844)

CMVD, coronary microvascular dysfunction.

#### Comparison of diagnostic time between CMVD_MDAS and commercial software

**Figure 8:**
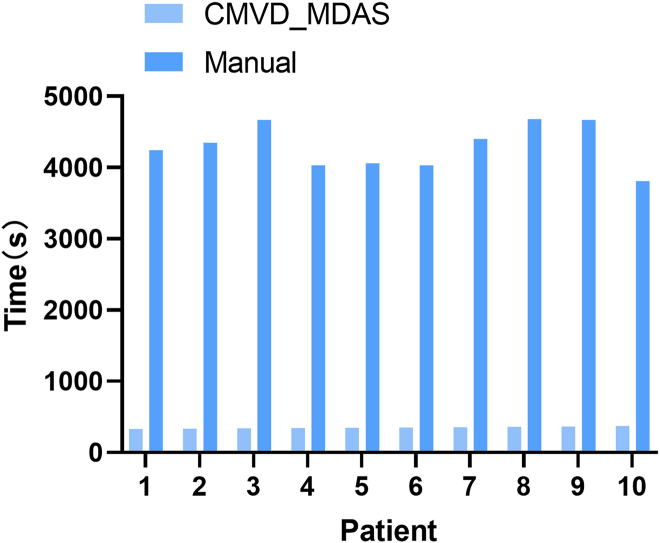
Software and CMVD_MDAS analysis of CMVD multimodal image duration

CMVD_MDAS enabled faster diagnosis compared to commercial software. In CMVD_MDAS, segmentation requires 0.21 s per image, while perfusion analysis, strain analysis, and classification take 0.18, 0.18, and 0.08 s, respectively. Because the latter three ran in parallel after segmentation, the total processing time per image was 0.39 s. We compared the analysis times for CMVD_MDAS and commercial software for 10 randomly selected external validation cases. CMVD_MDAS was significantly faster, with an average diagnostic time of 351 ± 13.16 s per patient (*p* < 0.001), as shown in Figure 8, where error bars indicate standard deviation. These results highlight the efficiency of CMVD_MDAS in delivering swift segment-level diagnoses from multimodal imaging data.

## Discussion

Explainable fusion of perfusion, mechanics, and metabolism yields clinically coherent segment-level decisions. This model enables precise myocardial segmentation in cardiac multimodal images from echocardiography, CMR, and PET/CT. It also performs qualitative and quantitative analyses of myocardial perfusion, strain, and metabolism. This model was tested and validated in both animal and patient cohorts. For CMVD diagnosis, multimodal imaging yields better accuracy than single-modality imaging. Additionally, CMVD_MDAS surpassed expert readers and commercial software while reducing processing time.

Recently, significant progress has occurred in DL using various cardiovascular imaging modalities. In echocardiography (Echo), lightweight convolutional and transformer-based networks such as EchoNet-Dynamic and UltraViT enable efficient real-time chamber segmentation and functional parameter estimation.^25^ In PET/CT, U-Net architectures enhanced by attention mechanisms improve myocardial perfusion quantification, lesion localization, and noise reduction, thereby enhancing certainty while decreasing dose.^26^ For MRI/CMR, models such as nnU-Net and Transformer-augmented UNets have achieved near-expert performance in myocardial segmentation and lesion detection, supporting precise functional assessment.^27^ In NMR spectroscopy, DL approaches optimize metabolite quantification and biomarker extraction, improving the detection of cardiovascular metabolic abnormalities.^28^ These advances have improved the accuracy and efficiency of image interpretation and highlight the promise of multimodal integration. Our CMVD_MDAS model builds upon these developments by integrating Echo, PET/CT, and CMR data to provide a comprehensive and interpretable diagnostic tool for coronary microvascular dysfunction.

In myocardial segmentation for cardiovascular disease diagnosis, image processing speed and segmentation accuracy are key concerns in clinical practice. AI has demonstrated advantages for both segmentation accuracy and speed, making it suitable for intelligent medical image analysis. In our segmentation model, we used a pyramid-style transformer encoder and introduced a PLD.^29^ PLD emphasizes local features and restricts the diffusion of attention, allowing the model to better capture local information in the input sequence and improving generalization ability and efficiency. The SSformer segmentation model in CMVD_MDAS outperformed Unet, Unet++, and nnUnet models in terms of segmentation accuracy for cardiac multimodal imaging data. Herein, the segmentation performance of MCE images was Dice = 0.92, IOU = 0.84, and HD = 22.86. The ability of our model to segment cardiac multimodal images surpasses the performance of U-net + bi-ConvLSTM^30^ and three-dimensional U-Net automated segmentation models^18^^,^^31^ introduced a universal medical segmentation model, MedSAM, which performed excellently in CMR segmentation, with a dice score exceeding 0.9. Similar to our results, it achieved comparable segmentation accuracy across modalities. Second, each image segmentation required 0.211 s, which was significantly faster than the segmentation speed of clinicians. Compared with general segmentation models, the U-Net structure and skip connection mechanism may achieve slightly quicker speeds in some cases.^32^ However, SSformer achieves better local feature extraction by incorporating a pyramid pooling module.

The diagnosis of viable myocardium is a key marker for guiding treatment plans and predicting the prognosis of CMVD patients. Therefore, the disease status of CMVD must be assessed from three perspectives: myocardial microcirculatory perfusion, myocardial mechanics, and myocardial viability. Abnormal microcirculatory blood flow in CMVD can lead to changes in myocardial wall motion and viability. Strain imaging (STI) can detect early systolic dysfunction in patients with microvascular lesions.^33^^,^^34^ ^18^F-FDG myocardial metabolic imaging is the reference standard for assessing viable myocardium, which is an essential indicator for determining the potential benefit of coronary revascularization. For myocardial contrast echocardiography (MCE) images, the traditional expert visual qualitative analysis method for diagnosing CMVD has an accuracy of 58%, a sensitivity of 41.0%, and a specificity of 83.0%.^35^ The sensitivity and specificity of PET/CT for the diagnosis of myocardial viability are 83% and 64%, respectively.^36^ In this study, a standard convolutional neural network (CNN) was used to predict normal/abnormal myocardial perfusion or metabolic segments for binary classification and to semi-quantitatively measure myocardial perfusion and myocardial strain. CMVD_MDAS integrates data on myocardial perfusion, mechanics, and viability obtained from MCE, CMR, and PET/CT.^37^ Using post-fusion techniques, the model combined qualitative and quantitative myocardial perfusion parameters to diagnose CMVD, achieving strong diagnostic performance: AUC of 0.79, sensitivity of 0.70, and specificity of 0.84. This approach addresses the challenges of long diagnostic times and low accuracy in clinical diagnosis of CMVD, thereby enabling precise diagnosis.

This study combines the LightGBM classification algorithm^38^ with SHAP explainability technology^39^ to construct a microcirculation function evaluation system with diagnostic advantages. As a clinically validated and efficient classifier, LightGBM integrates multidimensional features through a gradient boosting framework, offering superior diagnostic performance compared with traditional methods. SHAP summary plots showed that the three most critical diagnostic features included CMR_vision (myocardial tissue characteristics), MBF_rest (microcirculatory perfusion), and MCE_vision (perfusion functional characterization). CMR_vision reflects pathological changes in the myocardial tissue^40^ MBF_rest quantitatively assesses microvascular perfusion,^18^ and MCE_vision captures microcirculatory dysfunction via dynamic perfusion patterns. The clinical value of this system lies in its ability to provide radiologists with a feature contribution heatmap (red for positive effects/blue for negative effects) that enables rapid interpretation of decision logic for the model. Combining temporal trends in perfusion parameters facilitates a seamless transition from population-based feature analysis to individualized and precise diagnosis. This “algorithmic decision-visual validation” dual verification model enhances explainability and clinical applicability of CMVD diagnosis.

In conclusion, CMVD_MDAS delivered explainable, segment-level CMVD assessments with strong external discrimination and markedly faster processing than clinical software. The model achieved high diagnostic accuracy (AUC = 0.79), rapid processing (0.211 s per image), and robust interpretability using SHAP-based feature contribution maps. Compared with conventional visual assessment and commercial software, CMVD_MDAS demonstrated superior performance in terms of both diagnostic accuracy and efficiency. By establishing a closed-loop diagnostic framework that combines multimodal imaging, AI-based analysis, and explainability, this study addresses the current limitations of subjective interpretation and fragmented analysis, thereby providing a scalable and intelligent solution for CMVD assessment. Clinically, it enables radiologists to generate accurate and interpretable results in real time, thus supporting individualized treatment planning. Although limitations remain, including a relatively small external validation cohort, single-center data, variability in reference standards, and absence of healthy controls, this study lays the foundation for future multicenter validation, adoption of standardized benchmarks, and further optimization of cross-modal feature fusion to enhance diagnostic accuracy and clinical applicability.

### Limitations of the study

This study has several limitations. First, the model was trained using preclinical animal data, which differ from human clinical data in terms of species characteristics and imaging acquisition, potentially limiting direct clinical applicability. The benchmarks of training (pathological H&E) and external validation (PET/CT) cohorts were inconsistent, and the latter lacked a blank control group, which may have introduced label bias. Second, model performance decreased in clinical patients, reflecting limited adaptability to cross-domain data. Further optimization of transfer learning strategies is warranted. Third, clinical validation was constrained by a small, single-center sample with limited disease diversity and no pathological benchmarks, making multicenter, large-sample studies necessary. Fourth, the current post-fusion architecture of CMVD_MDAS, although robust, may lead to information loss between imaging modalities. Future work should explore deep-fusion approaches, such as cross-modal feature alignment. Fifth, multimodal imaging studies for CMVD remain scarce; owing to dataset limitations, including variability in image resolution, differences in acquisition protocols, and incomplete modality pairing, robust multimodal cross-validation could not be performed. Addressing these limitations will be a priority in future studies.

## Resource availability

### Lead contact

Further information and requests for resources should be directed to and will be fulfilled by the lead contact, Yuming Mu (mym1234@126.com).

### Materials availability

This study did not generate new unique reagents.

### Data and code availability

#### Data

The multimodal imaging data (US, CMR, and PET/CT) utilized in this study contain sensitive patient information and will not be made publicly available to protect patient privacy. Researchers with legitimate and qualified requests can contact the corresponding author, Dr. Mu, for data access upon completion of a formal data use agreement (DUA) and ethical approval.

#### Code

The underlying code for the Explainable-Enhanced AI model is not publicly available. However, it may be provided to qualified, non-commercial researchers upon reasonable request from the corresponding author.

#### Missing

All unique reagents, physical materials, and other items generated in this study are fully described within the STAR Methods and can be made available from the corresponding author upon reasonable request.

## Acknowledgments

This study was funded by the 10.13039/501100001809National Natural Science Foundation of China (32071459); the “Tianshan Talents” Programme for Cultivating High-level Talents in Medicine and Health Care (TSYC202301B002); Major Scientific Research Project Cultivation Program of 10.13039/501100004880Xinjiang Medical University (Project No. XYD2024ZX07); and Excellent Talent and Innovative Team Cultivation Program of the First Affiliated Hospital of Xinjiang Medical University (cxtd202424). This work is supported by Extreme Smart Analysis platform (https://www.xsmartanalysis.com/).

## Author contributions

Y.M. and J.C. initiated and supervised the project and provided the concept and design of the experiments; G.W., L.G., and S.L. developed the computational pipeline and analyzed the data; G.W., L.G., Y.Q., Y.W., and X.L. provided the clinical and pathological data curation from multiple medical centers; G.W., S.L., and Y.M. wrote the manuscript. All authors read and approved the final manuscript. G.W., L.G., and S.L. contributed equally to this work as the first authors.

## Declaration of interests

We declare no competing interests related to this study.

## STAR★Methods

### Key resources table

**Table:** 

REAGENT or RESOURCE	SOURCE	IDENTIFIER
**Experimental models: Organisms/strains**		

New Zealand White rabbit	Rabbit	Animal Experiment Center of Xinjiang Medical University

**Software and algorithms**		

Python (version = 3.11.4)	Python software	https://www.anaconda.com/products/distribution
3D-Slicer (version = 4.11.20210226)	3D-Slicer software	https://www.slicer.org/
SimpleITK (version = 2.0.2)	Python package	https://pypi.org/project/SimpleITK/
Torchvision (version = 0.9.0)	Python package	https://pypi.org/project/torchvision/
Scikit-image (version = 0.18.3)	Python package	https://pypi.org/project/scikit-image/
PyTorch (version = 1.8.0)	Python package	https://pytorch.org/get-started/previous-versions/
Scikit-learn (version = 0.24.2)	Python package	https://pypi.org/project/scikit-learn/
SciPy (version = 1.7.3)	Python package	https://pypi.org/project/scipy/
SSFormer	GitHub	https://github.com/Qiming-Huang/ssformer
UNet	GitHub	https://github.com/milesial/Pytorch-UNet
UNet++	GitHub	https://github.com/MrGiovanni/UNetPlusPlus
nnUNet (version = 2.2)	GitHub	https://github.com/MIC-DKFZ/nnUNet
Extreme Smart Analysis platform	Analysis platform	https://www.xsmartanalysis.com/
R software(version = 4.2.3)	R software	https://www.r-project.org/
CVI 42(version = 5.17)	CMR Analysis software	https://www.circlecvi.com/cardiac-mr
Qlab workstation (version = 13.0)	philip Analysis software	Philips
Xeleris functional imaging workstation	MMI Analysis software	GE Healthcare
Pair software (version = 2.7)	Segment software	RayShape

### Experimental model and study participant details

This retrospective study was conducted in accordance with protocols approved by the Institutional Review Board of the First Affiliated Hospital of Xinjiang Medical University (approval no. K2020302-27) and the Experimental Animal Research Center of Xinjiang Medical University (IACUC-20210725-15). All procedures involving laboratory animals complied with the Declaration of Helsinki and the Guide for the Care and Use of Laboratory Animals. Written informed consent was obtained from all participants before data collection.

The sample size in this study was empirically estimated based on the desired precision (confidence interval width) of diagnostic performance metrics, such as sensitivity and specificity, requiring 196 positive samples and 139 negative samples. Ultimately, 944 independent myocardial segments (over 310,000 multimodal images) were included.

A rabbit model of CMVD was established (Figure S1)^40^ using pathological H&E staining as the benchmark for diagnosis. Ten normal male rabbits served as controls, and 27 male rabbits underwent induction of coronary microvascular dysfunction via ultrasound-guided intraventricular sodium laurate injection. Multimodal imaging, including MCE, CMR, and ^18^F-FDG PET/CT, was performed on postoperative day 3. H&E staining was performed for myocardial pathology in all 37 rabbits, with positive segments defined by inflammatory cell infiltration, microthrombus formation, or fibrosis on the pathology slides. Overall, 111 slides were processed into 592 myocardial segments using the 16-segment model proposed by the American Society of Echocardiography (Figure S2). MCE-echo, CMR, and ^18^F-FDG PET/CT images of 37 rabbits were matched to corresponding histopathological sections following a standardized image acquisition and data analysis protocol. The pathology reports were provided by an expert with 15 years of experience. Clinical images from 22 patients (16 were male, and 6 were female) were processed into 16 myocardial segments and used as an external validation set to assess the generalizability of the CMVD-MDAS model.

### Method details

#### Inclusion and exclusion criteria for CMVD patients

To define the study cohort, we established specific inclusion and exclusion criteria.

A total of 22 clinically diagnosed INOCA patients (16 male, 6 female) were enrolled, all of whom had undergone coronary angiography or coronary CTA showing no more than 50% coronary stenosis. All patients received 18F-FDG PET/CT myocardial metabolic imaging, CMR, and MCE assessments. The PET/CT report served as the reference standard for diagnosing myocardial segments in these patients. Exclusion criteria consisted of the following: (i) myocardial infarction, valvular disease, hypertrophic cardiomyopathy, or other conditions potentially affecting the study outcomes; (ii) major diseases including cancer; (iii) diabetes or poorly controlled glycemic levels; (iv) peripheral vascular diseases such as aortic dissection; (v) poor image quality.

#### Establishment of the rabbit CMVD disease model

A rabbit CMVD model was established to recapitulate human disease pathology. Following anesthesia and immobilization, the right common carotid artery of the rabbit was isolated, and a 6Fr (2.0 mm) balloon catheter was introduced. Under ultrasound guidance, the balloon was advanced through the right internal carotid artery and brachiocephalic trunk into the ascending aorta. The balloon was then inflated to transiently obstruct blood flow in the ascending aorta for approximately 20 s. Concurrently, sodium laurate was injected into the heart cavity via an apical approach under ultrasound guidance. This compound induces endothelial cells injury, leading to subsequent coronary microcirculatory dysfunction. This modeling procedure recapitulates the pathological progression observed in patients^40^ as shown in Figure S1.

#### Standardized data acquisition – Cardiovascular magnetic resonance (CMR)

CMR images were acquired using a standardized protocol on a 3.0 T scanner. CMR scans were acquired on a 3.0 T S Skyra MRI scanner using a knee coil. The imaging protocol, adapted from Patel et al.^41^ encompassed both resting and stress states, as illustrated. The specific parameters for each sequence are detailed below.

(1) **Black-Blood Sequence:** FSE, axial orientation, **echo time (TE)** = 1.74msec, repetition time **(TR)** = 752.00 msec, field of view (FOV) = 160 mm × 160 mm, matrix size = 160 × 160, slice thickness = 4.0 mm, number of slices = 10, averages = 32, voxel size = 0.8 × 0.8 × 4.0 mm^3^, flip angle = 10°, and scan time = 241 s.
(2) **Rest cine Sequence:** FSE, axial orientation, echo time (TE) = 1.71 msec, repetition time (TR) = 47.28 msec, field of view (FOV) = 196 mm × 196 mm, matrix size = 196 × 196, slice thickness = 4.0 mm, number of slices = 8, averages = 3, voxel size = 0.9 × 0.9 × 4 mm^3^, flip angle = 65°, and scan time = 250 s. Signal mode:ECG/Retro.
(3) **Rest First-Pass Perfusion Sequence:** FSE, axial orientation, echo time (TE) = 1.27 msec, repetition time (TR) = 186.49  msec, field of view (FOV) = 220 mm × 220mm, matrix size = 220 × 220, slice thickness = 6.0 mm, number of slices = 3, averages = 3, voxel size = 1.1 × 1.1 × 6.0 mm^3^, flip angle = 10°, and scan time = 50 s.

##### Data Analysis

The myocardial left ventricular radial strain parameters and blood flow perfusion parameters were analyzed using the CVI 42 software.^42^

#### Standardized data collection — Myocardial contrast echocardiography

MCE was performed to quantitatively assess myocardial microcirculation perfusion. Following anesthesia the rabbit and electrocardiogram connection, intravenous access was established via the left ear vein for ultrasound contrast agent infusion (Sonovue, Bracco, Italy). Images were acquired using a Philips EPIQ 7 C ultrasound system equipped with an S5-1 phased-array transducer, with settings switched to “Contrast” mode using the following parameters: an image depth set to 50 mm, Gain adjusted to 55 ± 5%, Focus placed at the mitral valve level, Dynamic range set to 50 dB, and Mechanical index (MI) maintained at 0.18, These settings remained consistent throughout the examination. Short-axis images of the mitral valve, papillary muscles, and apex were sequentially captured, each image acquisition lasting 10 s. All data were stored in DICOM format for subsequent offline analysis.

##### Data Analysis

Quantification assessment of myocardial microcirculation perfusion was conducted offline using Philips Qlab software (Qlab 13.3; Philips Medical System, Andover, MA, USA). End-systolic phase myocardial segments were selected as region of interest (ROI), excluding structures such as the left ventricular cavity and pericardium. The peak signal intensity of the myocardium (A) was derived using a power law equation, representing blood volume, while the slope of the curve (*β*) indicated blood flow velocity. Perfusion parameters, including resting-state blood volume (A), perfusion velocity (*β*), and myocardial blood flow (MBF = An ∗*β*), were obtained for each myocardial segment.

#### Standardized data collection—^18^F-FDG PET/CT

PET/CT imaging was conducted to evaluate myocardial metabolic activity. ^18^F-FDG PET/CT imaging was conducted on a GE Discovery PET/CT system. A dosage of 0.1 mCi/kg of 18F-fluorodeoxyglucose was administered intravenously, and image acquisition commenced 60 min after injection. Subjects were positioned supine and connected to an ECG gating device, followed by an 8-min static scan. Three-dimensional images were acquired with the following parameters: 40 mm detector coverage, 2.5 mm spiral slice thickness, a pitch of 0.984:139.37 mm/rot, 0.6-s rotation time, 140 kV voltage, and 150 mA current, using a 50 cm dynamic field of view. Images reconstructed employed GE Healthcare’s 3D VUE point algorithm with ordered subset expectation maximization algorithm, with a 35 cm diameter and 128 × 128 lateral matrix. CT attenuation correction was performed during reconstruction, using 4 iterations and 8 subsets. This procedure generated short-axis, vertical long-axis, and horizontal long-axis tomographic images of the left ventricle, with consistent parameters maintained across all scans and reconstructions.

##### Data Analysis

To quantitatively assess myocardial metabolic activity, standardized uptake values (SUV_bw_) and segment scores (PET_score_) were used. The Xeleris Functional Imaging Workstation was employed to locate and read the ^18^F-FDG uptake SUV_bw_ values for each myocardial segment in the coronary, sagittal, and axial planes.

#### ASE 16-region segmentation method

The left ventricle was divided according to the standardized ASE 16-segment model. The American Society of Echocardiography (ASE) introduced a 16-segment model for dividing the left ventricular myocardium.^24^ This model partitions the ventricular myocardium into three regions: basal, mid-cavity, and apical rings. The height of each ring approximately one-third of the total ventricular length. Anatomically, the basal ring spans from the mitral valve annulus to the tips of the papillary muscles, the mid-cavity ring extends from the papillary muscle tips to their bases, and the apical ring comprises the myocardium distal to the papillary muscle bases, as illustrated in Figure S2.

#### Image annotation

Manual annotation of multimodal images was performed to create the ground truth for model development. Image annotation was performed on multiple imaging datasets to develop and evaluate the CMVD_MDAS model for multimodal myocardial segmentation. Overall, 1,539 echocardiographic images, 1,000 cardiac magnetic resonance images, and 394 PET/CT myocardial metabolic images were manually annotated. The annotations were completed using Pair software (version 2.7; RayShape, Shenzhen, China).

#### Image preprocessing

Raw images underwent preprocessing to standardize input data. Prior to being input into CMVD_MDAS, raw multimodal images underwent preprocessing, including cropping, resizing, normalization, rotation, contrast/brightness/sharpness adjustment, and random Gaussian blur (Figure S3). The images were cropped to 400 × 400 pixels. These steps were performed using SimpleITK (version 2.1.1). Myocardial tissue features were extracted from multimodal images using the SSformer segmentation model.

#### Model development

This study developed a CMVD_MDAS diagnostic model using multimodal myocardial images as inputs and yielding CMVD probability and binary classification results as outputs.

#### Multimodal cardiac segmentation task

The SSformer framework was optimized for accurate myocardial segmentation. The SSformer segmentation framework, based on a transformer architecture, was optimized for accurate myocardial segmentation. The model incorporates a pyramidal transformer encoder to enhance its generalizability and employs a progressive local decoder (PLD) to emphasize local features and limit attention diffusion (Figure 1B).

Design of the Pyramid Transformer (PVTv2) encoder is inspired by the architecture of PVTv2 and Segformer. These models replace traditional Transformer position encoding (PE) operations with convolutional operations to maintain spatial information consistency, offering exceptional performance and stability. The Progressive Local Decoder (PLD) is a novel multi-stage feature aggregation decoder for feature pyramids. PLD includes the Local Emphasis (LE) module and Stepwise Feature Aggregation (SFA) module. The LE module focuses the model’s attention on key details, while the SFA structure effectively limits the model’s attention to fine-tuning critical regions, minimizing irrelevant distractions.

#### Qualitative and quantitative classification tasks

Segmentation results were further processed for classification and quantitative analysis. After obtaining the segmentation results, we applied a 7-layer CNN classification model with pooling and fully connected layers to produce predictions. Using myocardial contrast echocardiography and cardiac magnetic resonance first-pass perfusion imaging, time–perfusion curves were fitted to analyze myocardial microcirculation perfusion characteristics. Left ventricular radial strain features were analyzed using dynamic cine imaging sequences of myocardial contrast echocardiography and cardiac magnetic resonance imaging.

We selected a lightweight CNN architecture because it provides competitive performance with high efficiency, supports transparent, interpretable decision-making for clinical adoption, and enables practical deployment in resource-constrained healthcare environments.

The core idea of a CNN is to extract features from input data through convolutional operations. It comprises multiple convolutional, pooling, and fully connected layers. CNNs can automatically learn features from images or sequence data, making them highly effective in handling complex tasks. Pooling reduces the size of feature maps and lowers computational complexity while retaining essential information for processing in subsequent layers. The fully connected layer in a neural network performs a linear transformation that maps input features to the outputs.

#### Computer vision classification

The output of the ssformer segmentation model, which includes the segmented myocardium and myocardial segments, is processed through a 7-layer CNN classification model with pooling and linear layers to obtain the final classification results.

CNNs (Convolutional Neural Networks) extract and learn features from data through components such as convolutional layers, pooling layers, and fully connected layers. Pooling is a technique in CNNs that reduces the data’s dimensionality and computational load by sampling the feature maps outputted by convolutional layers, while preserving key information. Linear transformation refers to the process of linearly combining inputs using weight matrices and bias vectors.

Overall, the model consists of a feature extractor built from convolutional layers and a fully connected network for classification. Together, they perform feature extraction and binary classification tasks on image data.

#### Quantitative perfusion analysis

##### MCE quantitative perfusion analysis

According to the guidelines from the American Society of Echocardiography^43^ MCE quantitative perfusion analysis involves obtaining a time-intensity curve, which is then fitted to a single exponential function:

Y = *A*×(1-e*^-βt^*)

In this context, *Y* represents the myocardial ROI intensity at time *t* following the “Flash” pulse, while *A* indicates the intensity of the myocardial ROI during the plateau phase. The slope of the curve reflects the mean microbubble replenishment rate, denoted as *β*. Myocardial blood flow (MBF) can be described as: *MBF* = *A*×*β*.

The perfusion values of rest MCE obtained through AI-based quantitative perfusion methods includeA_rest、*β*_res, MBF_rest.

#### CMR quantitative perfusion analysis

The voxel concentration curves extracted from the myocardial segments are used for perfusion quantification through tracer kinetics modeling, with hierarchical Bayesian inference to estimate the kinetic parameters.

RMU = Relative enhancement rate of the myocardium ÷ Relative enhancement rate of the blood pool.

#### Strain analysis

Left ventricular radial strain (RS) refers to the thickening and thinning motion of the myocardium in the short-axis direction, perpendicular to the epicardium toward the center of the heart cavity. It reflects radial myocardial deformation corresponding to the thickening during systole, with strain values typically being positive. Radial strain of the left ventricular myocardium is calculated using the end-diastolic and end-systolic endocardial and epicardial segmentation results from MCE and CMR cine sequence video data.

The formula for strain is: *Strain=(L1-L0)/L0*.

*L0* is the initial length of the myocardial segment at end-diastole (natural state), *L1* is the length of the myocardial segment at a given time (e.g., at end-systole).

#### Establishment of multimodal fusion diagnostic model

A fusion model was built by integrating multiple features and classifiers. The classification probability, myocardial microcirculation perfusion metrics, and myocardial strain analysis values were obtained using qualitative and quantitative methods. Feature selection was performed using the LassoCV method, and variable importance analysis was conducted using Lasso regression. Multiple machine learning models, including logistic regression, LGBM, random forest, and AdaBoost classifiers, were employed for classification. The optimal model was selected based on its performance in the training and internal validation sets.

In the second phase, both qualitative and quantitative methods are used to derive binary classification prediction probabilities, myocardial microcirculation perfusion metrics, and myocardial strain analysis values. Various machine learning models, including Logistic Regression, LGBM Classifier, Random Forest Classifier, and AdaBoost Classifier, are employed to perform the data sample classification task. The best model is selected based on performance in the training set and internal validation set.

The parameter values for each model are selected as follows:

**LogisticRegression Model Parameters:** C: 1.0; max_iter: 100; penalty: l2;tol: 0.0001.

**LGBMClassifier Model Parameters:** boosting_type: gbdt;learning_rate: 0.1;max_depth: −1;n_estimators: 100;num_leaves: 31.

**RandomForestClassifier Model Parameters:** Criterion: gini;max_depth: None;min_impurity_decrease: 0.0;n_estimators: 20.

**AdaBoostClassifier Model Parameters:** learning_rate: 1.0;n_estimators: 50.

#### Training parameters

Models were trained with specified parameters and optimization strategies. The model was implemented in PyTorch (version 1.8.0) and accelerated using NVIDIA Tesla A100 and RTX 3090 GPUs. Training was conducted with the AdamW optimizer with an initial learning rate of 0.0001, a decay rate of 0.1, and a decay period of 50 epochs for a total of 150 epochs. In some training phases, to improve model generalization, the Adam optimizer (β_1_ = 0.9, β_2_ = 0.999) was used with a cosine annealing learning rate scheduler initialized at 2 × 10^−5^. The batch size was set to 16.

All U-Net methods used 3 × 3 × 3 convolution kernels with five pooling operations along the z- and y axes and four along the x axis. The loss function combined Dice and BCE losses; specifically, the main classification task used cross-entropy loss, whereas the auxiliary segmentation task employed Dice loss to improve boundary prediction accuracy. During training, the images were cropped to 400 × 400 pixels, and data augmentation included random flipping, scaling, rotation, dilation, and blurring.

Variable importance analysis was performed using LASSO regression with these parameters: cv = 5, max_iter = 1,000, tol = 0.0001, and alpha = 0.0003.

#### Comparison with radiologists and medical analysis software

CMVD_MDAS performance was compared against clinical experts and commercial software. To clearly assess the clinical applicability and potential advantages of CMVD_MDAS, we compared its diagnostic performance with that of senior radiologists and widely used commercial medical analysis software (Qlab 13.0, CVI 42 for cardiac magnetic resonance imaging, and Xeleris Functional Imaging Workstation for PET/CT). The comparison was conducted on both the internal validation set (animal cohort) and the external test set (clinical patient cohort).

The radiologists, blinded to the ground truth, evaluated all myocardial segments independently using multimodal images according to routine diagnostic workflows. Commercial software analyses were performed according to the standard protocols of each manufacturer for the respective modality.

The objective of this comparison was to determine whether CMVD_MDAS could achieve diagnostic accuracy, sensitivity, and specificity comparable to or exceeding those of expert human readers and current clinical software, thereby demonstrating its potential for reliable and efficient diagnosis of CMVD in clinical settings.

#### Model evaluation metrics

Model performance was evaluated using a comprehensive set of metrics. We evaluated multimodal image segmentation performance using seven metrics: accuracy, recall, F1 score, Dice coefficient, intersection over union (IoU), Hausdorff distance, and area under the receiver operating characteristic (ROC) curve (AUC). Multimodal image classification performance was assessed using the following metrics: AUC, sensitivity, specificity, accuracy, F1 score, positive predictive value (PPV), negative predictive value (NPV), kappa value, confusion matrix, calibration curve, decision curve, and KS statistic plot. SHapley Additive exPlanations (SHAP) value plots were used to interpret the effects of individual features on the model output in the machine-learning framework.

### Quantification and statistical analysis

Statistical analyses were performed to ensure the robustness of the findings. Categorical data were analyzed using the chi-square test, while continuous variables were assessed using the *t* test if normally distributed or the nonparametric rank-sum test. Factors identified as significant in univariate analysis were included in a binary logistic regression model, with stepwise regression applied for further selection of meaningful variables. The DeLong test was used to compare differences between ROC curves. Machine learning models, including logistic regression, LGBM classifier, random forest classifier, and AdaBoost classifier, were constructed using the Smart Analysis platform. Statistical analyses were conducted using SPSS version 26.0 and Smart Analysis platform. Feature selection, variable importance analysis, and the development and validation of binary classification models were performed using R version 4.2.3 and Python version 3.11.4.
