# Supplementary material for: Explainable-enhanced AI for diagnosing coronary microvascular dysfunction with multimodal imaging
Source: iScience. 2025 Nov 19;28(12):114101. doi: 10.1016/j.isci.2025.114101 (PMC12721181; doi:10.1016/j.isci.2025.114101)
Supplement: Document S1. Figures S1–S5 and Tables S1 and S2 [file mmc1.pdf]

iScience, Volume *28*

Supplemental information

Explainable-enhanced AI for diagnosing coronary microvascular dysfunction with multimodal imaging

Guodong Wang, Lina Guan, Shiyu Li, Yongde Qin, Yunling Wang, Xiaohong Li, Jie Chen, and Yuming Mu

Supplementary Figures

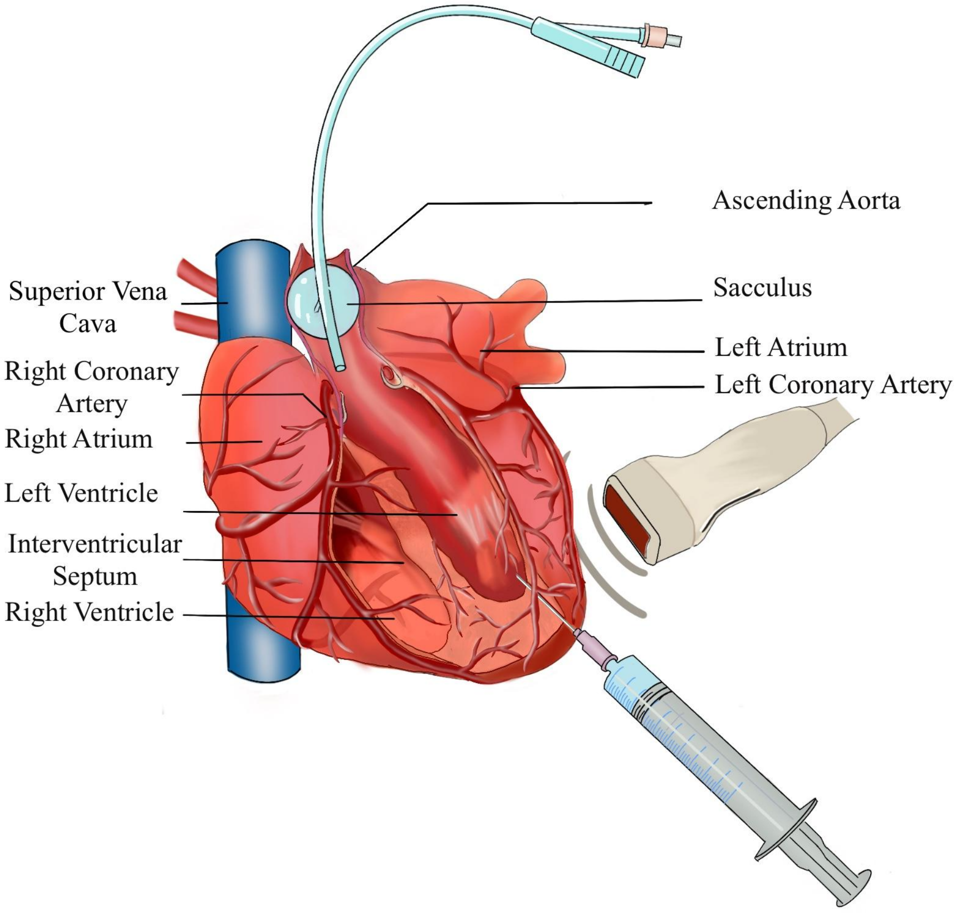

Figure S1. Schematic of CMVD Rabbit Animal Model Construction

The model is established using ultrasound-guided ascending aortic blood flow occlusion and intracavitary drug injection.

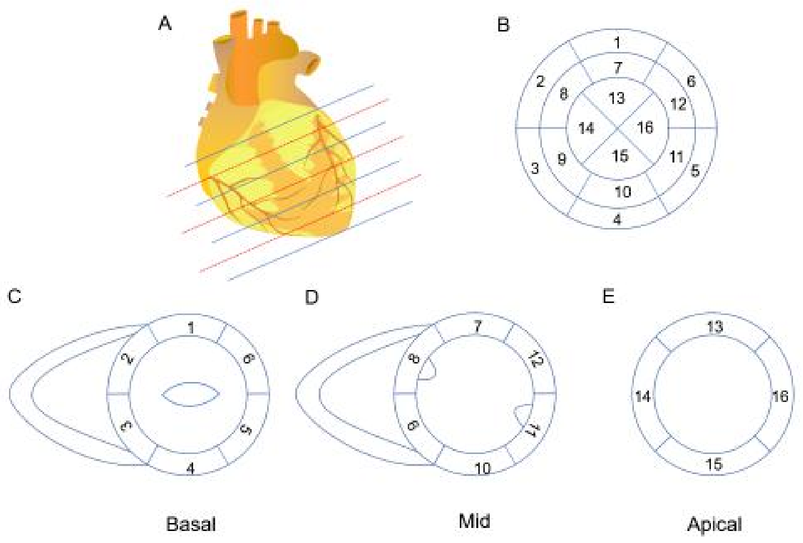

Figure S2. Schematic diagram of the 16-segment segmental approach to the left ventricular myocardium

1. Basal anterior; 2. Basal anteroseptal; 3. Basal inferoseptal; 4. Basal inferior; 5. Basal inferolateral; 6. Basal anterolatera; 7. Mid anterior; 8. Mid Anteroseptal; 9. Mid Inferoseptal; 10. Mid Inferior; 11. Mid Inferolateral; 12. Mid anterolatera; 13. Apical anterior; 14. Apical septal; 15. Apical inferior; 16. Apical latera

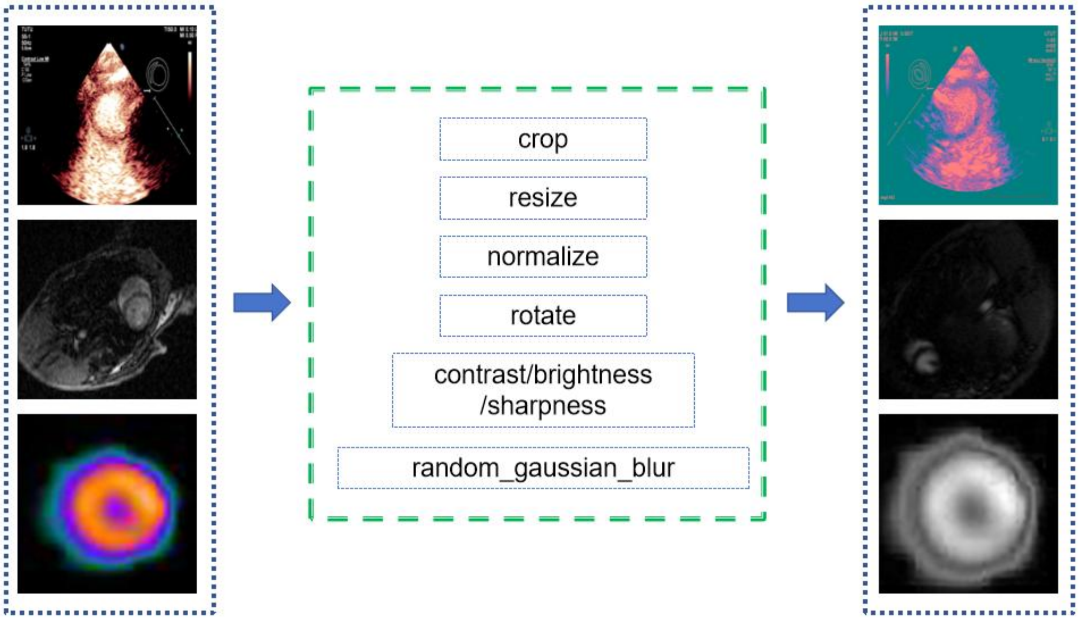

Figure S3. Image pre-processing for multimodal images

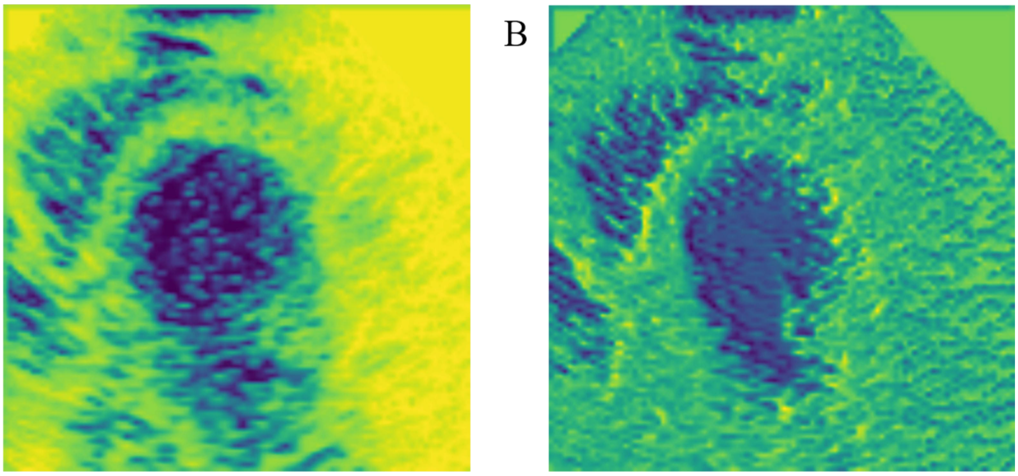

A Myocardial attention， High

B Myocardial attention， Low

Figure S4. Visualisation of myocardial segmentation

The masformer model gives high attention to the myocardial region and low attention to the ventricles. Yellow represents high attention and blue represents low attention.

LogisticRegression **LGBMClassifier** **RandomForestClassifier** **AdaBoostClassifier**

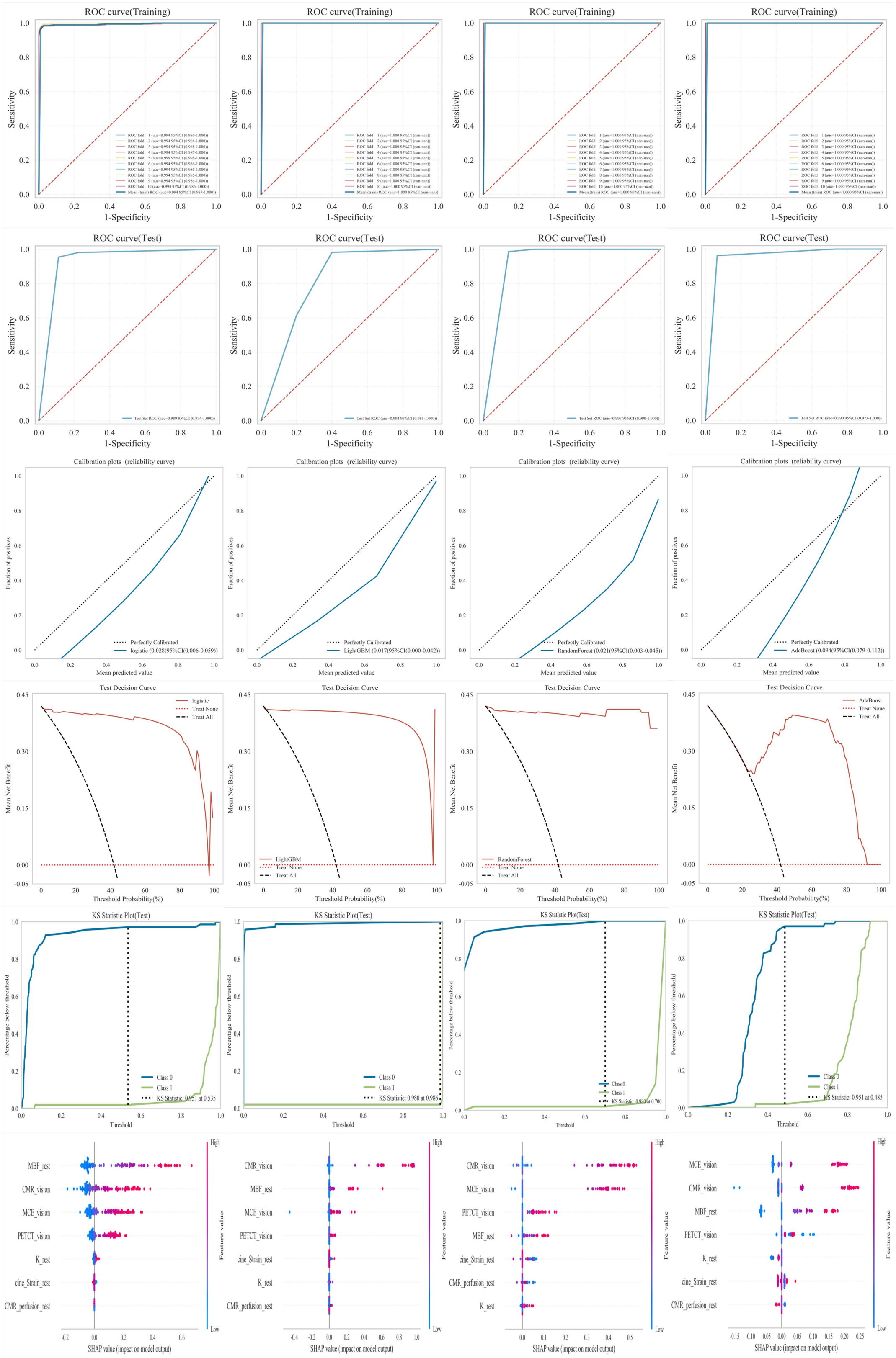

Figure S5 Performance comparison of various classification models

The first row presents the ROC curve for the training set, while the second row displays the ROC curve for the test set, illustrating the model's discrimination ability

in both datasets. The third row shows the calibration curve, assessing the agreement between predicted and actual outcomes. The fourth row depicts the decision curve, evaluating the clinical utility of the model. The fifth row presents the KS statistic plot, which measures the model's ability to distinguish between positive and negative cases. Finally, the sixth row features the SHAP value plot, providing insights into the contribution and importance of each factor in the model's predictions.

Supplemenary Tables

Table S1. Segmentation effect evaluation indices of different segmentation models

Datatype	Model	precise	recall	dice	Fmeasure F	iou	hausdorff_95
MCE segment	ssformer	0.9146*	0.8628*	0.9153*	0.9153*	0.8437*	22.858*
	Unet	0.7086	0.7098	0.7163	0.7011	0.6912	46.77
	Unet+	0.7954	0.7510	0.7814	0.7896	0.7455	32.13
	nnUnet	0.8039	0.7478	0.7968	0.7947	0.7506	33.09
CMR segment	ssformer	0.8861*	0.8716*	0.8736*	0.8861*	0.8123*	15.42*
	Unet	0.6911	0.6871	0.7031	0.7099	0.6788	40.66
	Unet++	0.7566	0.7326	0.7611	0.7834	0.7239	34.12
	nnUnet	0.7888	0.7649	0.7830	0.7910	0.7326	29.59
PET/CT segment	ssformer	0.8813*	0.8201*	0.8007*	0.8502*	0.7923*	18.30*
	Unet	0.7991	0.7456	0.7201	0.7621	0.7012	44.32
	Unet++	0.7341	0.7765	0.7214	0.7623	0.7032	46.13
	nnUnet	0.6734	0.6397	0.6789	0.6801	0.6543	68.19

Table S2. Comprehensive Evaluation of Model Performance

Cohort	Model	AUC(95%CI)	ACC	SEN	SPE	PPV	NPV	F1 Score	Kappa
Train	logistic	0.993 (0.986-1.000)	0.98	0.975	0.984	0.977	0.982	0.976	0.959
	LightGBM	1.000 （NaN-NaN）	0.998	0.996	1.0)	1.0	0.997	0.998	0.996
	RandomForest	1.000（NaN-NaN）	0.997	0.992	1.0	1.0	0.994	0.996	0.993
	AdaBoost	1.000（NaN-NaN）	0.998	0.996	1.0	1.0	0.997	0.998	0.996
Internal test	logistic	0.992 （NaN-NaN）	0.978	0.976	0.979	0.973	0.984	0.974	0.955
	LightGBM	0.994 （NaN-NaN）	0.98	0.952	1.0	1.0	0.967	0.975	0.958
	RandomForest	0.991 （NaN-NaN）	0.981	0.964	0.994	0.992	0.976	0.977	0.962
	AdaBoost	0.990 （NaN-NaN）	0.975	0.96	0.985	0.981	0.973	0.969	0.948
External test	logistic	0.735（0.68-0.789）	0.705	0.744	0.683	0.564	0.829	0.641	0.398
	LightGBM	0.79（0.737-0.844）	0.793	0.704	0.841	0.71	0.838	0.707	0.546
	RandomForest	0.767（0.711-822）	0.75	0.76	0.744	0.621	0.849	0.683	0.48
	AdaBoost	0.801（0.75-0.851）	0.767	0.736	0.784	0.652	0.844	0.692	0.506
